# Progressive bladder remodeling due to bladder outlet obstruction: a systematic review of morphological and molecular evidences in humans

**DOI:** 10.1186/s12894-018-0329-4

**Published:** 2018-03-09

**Authors:** Ferdinando Fusco, Massimiliano Creta, Cosimo De Nunzio, Valerio Iacovelli, Francesco Mangiapia, Vincenzo Li Marzi, Enrico Finazzi Agrò

**Affiliations:** 1 Dipartimento di Neuroscienze e Scienze Riproduttive ed Odontostomatologiche, Università Degli Studi Di Napoli Federico II, Via Pansini, 5, 80131 Naples, Italy; 2 Dipartimento di Urologia, Ospedale Sant’Andrea, Università Degli Studi di Roma “La Sapienza”, Rota, Italy; 3 Dipartimento di Medicina Sperimentale e Chirurgia, Università Degli Studi di Roma “Tor Vergata”, Roma, Italy; 4 Dipartimento di Urologia, Ospedale Careggi, Università Degli Studi di Firenze, Firenze, Italy

**Keywords:** Bladder outlet obstruction, Bladder remodeling, Systematic review

## Abstract

**Background:**

Bladder outlet obstruction is a common urological condition. We aimed to summarize available evidences about bladder outlet obstruction-induced molecular and morphological alterations occurring in human bladder.

**Methods:**

We performed a literature search up to December 2017 including clinical and preclinical basic research studies on humans. The following search terms were combined: angiogenesis, apoptosis, bladder outlet obstruction, collagen, electron microscopy, extracellular matrix, fibrosis, hypoxia, histology, inflammation, innervation, ischemia, pressure, proliferation, remodeling, suburothelium, smooth muscle cells, stretch, urothelium.

**Results:**

We identified 36 relevant studies. A three-stages model of bladder wall remodeling can be hypothesized involving an initial hypertrophy phase, a subsequent compensation phase and a later decompensation. Histological and molecular alterations occur in the following compartments: urothelium, suburothelium, detrusor smooth muscle cells, detrusor extracellular matrix, nerves. Cyclic stretch, increased hydrostatic and cyclic hydrodynamic pressure and hypoxia are stimuli capable of modulating multiple signaling pathways involved in this remodeling process.

**Conclusions:**

Bladder outlet obstruction leads to progressive bladder tissue remodeling in humans. Multiple signaling pathways are involved.

## Background

Bladder outlet obstruction (BOO), clinically defined as high-pressure/low-flow micturition pattern at urodynamic investigations, is a common urological condition in humans with benign prostatic obstruction (BPO) being the most frequent causative factor. It represents a key pathophysiological link between benign prostate enlargement (BPE) and lower urinary tract symptoms (LUTS) [1–3]. Besides symptoms, BOO can also lead to progressive tissue remodeling of the bladder and of the upper urinary tract with subsequent serious functional impairments [1–4]. Based on the results from studies on animal models exposed to experimental partial outlet obstruction, the remodeling of the bladder involves the modulation of several signaling pathways as well as histological alterations occurring in almost all cellular compartments [5]. These changes are described to progress through three sequential stages: hypertrophy, compensation and decompensation [6]. In the hypertrophy stage, mechanical stress activates early signals that mediate bladder wall hypertrophy. At the same time, due to the occurrence of focal area of hypoxia, angiogenesis is stimulated thus enabling blood flow to increase relative to bladder mass [6]. In the compensated stage bladder growth and angiogenesis stop. At some point, if obstruction persists, the bladder shifts to a decompensated state as a result of cyclical ischemia-reperfusion injury occurring during the micturition phase that leads to the activation of pathways involved in the progressive loss of smooth muscle, deposition of extracellular matrix and neuronal loss [6]. The duration of these stages varies considerably according to the experimental models and is unpredictably. Data about the reversibility of these alterations are also lacking. *H*uman detrusor differs significantly from animal models and most of the species used for research don’t suffer from naturally occurring outflow obstruction [7]. *The aim of the present study was to summarize available evidences about BOO-induced morphological and molecular alterations occurring in the various compartments of human bladder.*

## Methods

We performed a systematic review using the Preferred Reporting Items for Systematic Reviews and Meta-Analyses Statement as a guideline in the development of the study protocol and the report of the current study [8]. In December 2017 we used the National Library of Medicine PubMed search engine, the Scopus database, and the ISI Web of Knowledge official website to search for all published studies evaluating morphological and molecular alterations involved in BOO-induced bladder wall remodeling. The following search terms were combined: angiogenesis, apoptosis, bladder outlet obstruction, collagen, electron microscopy, extracellular matrix, fibrosis, hypoxia, histology, inflammation, innervation, ischemia, pressure, proliferation, remodeling, suburothelium, smooth muscle cells, stretch, urothelium. We included publications that met the following criteria: reporting original in vitro and in vivo research; English language; human studies. Reference lists in relevant articles and reviews were also screened for additional studies.

## Results

**Fig. 1:**
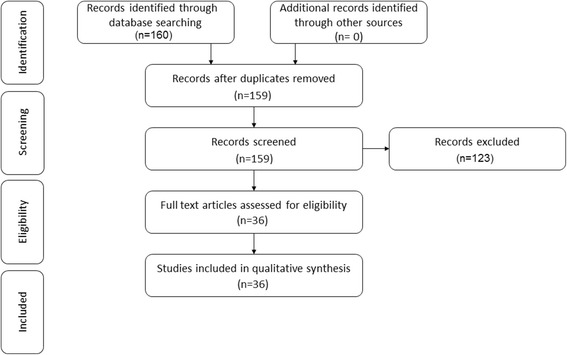
Preferred Reporting Items for Systematic Reviews and Meta-analysis (PRISMA) flowchart

The search strategy revealed a total of 159 results. Screening of the titles and abstracts revealed 44 papers eligible for inclusion. Further assessment of eligibility, based on full-text papers, led to the exclusion of 8 papers. This left 36 papers meeting our criteria for inclusion [9–44] (Fig. 1).

### Alterations occurring in the urothelium and suburothelium

We identified one study describing BOO-induced alterations occurring in the context of urothelium and suburothelium [9]. Bladder biopsies were obtained from 33 men with urodynamic proven BOO and 10 control subjects. Authors demonstrated the occurrence of significant urothelial dysfunction and alterations of urothelial signaling and sensory transduction pathways in patients with BOO. In detail, the following alterations were found: significantly lower expression of the adhesion protein E-cadherin and of the muscarinic receptor M3, and significantly higher expression of the purinergic receptor P2X3 and of the muscarinic receptor M2. Interestingly, patients with detrusor underactivity had a significantly lower expression of E-cadherin and of inducible nitric oxide synthase as well as a significantly higher expression of β3 adrenoreceptors than patients with detrusor overactivity/hypersensitive bladder. Clinically, lower expression of E-cadherin was associated with lower voided volumes thus suggesting a more severe or decompensated status with more severe urothelial dysfunction. Alterations found in the suburothelium of BOO patients included: inflammation, with a significantly increased level of activated mast cells, and increased apoptosis.

### Alterations occurring in the detrusor muscle: morphological aspects

The detrusor muscle represents the bladder compartment more extensively investigated. Studies describing BOO-induced morphological alterations occurring in the detrusor are summarized in Table 1. Significant alterations involving both smooth muscle cells (SMCs) and extracellular matrix (ECM) have been demonstrated using light and electron microscopy studies.

**Table 1:** Summary of studies describing BOO-induced detrusor morphological alterations

Author, year	Study design	Subjects in the case group (n)	Main findings
Gosling, 1980 [15]	Case control	9	• ↑ intrafascicular and interfascicular collagen • ↓ SMCs diameter
Gilpin, 1985 [10]	Case control	14	• ↑connective tissue infiltration of some smooth muscle bundles in 12/14 patients • ↑ SMCs mean profile area
Elbadawi, 1993 [12]	Case control	7	• ↓ intermediate cell junctions • ↑intrafascicular collagen and elastic fibers • ↑ SMCs hypertrophy • ↑ SMCs and axons degeneration in patients with impaired detrusor contractility
Inui, 1999 [19]	Case control	26	• Significant positive linear relationship between C/M and estimated bladder weight in patients with bladder weight ≥ 60 g • ↑ C/M in patients with bladder weight ≥ 60 g
Tse, 2000 [13]	Case control	9	• Myohypertrophy pattern in all cases
Brierly, 2003 [14]	Case control	12	• Myohypertrophy pattern in 8/10 BOO patients. • Degenerative pattern in 4/10 BOO patients.
Holm, 2003 [22]	Case control	25	• Significant correlation between intra- and interfascicular elastin and BOO degree
Mirone, 2004 [15]	Case control	36	• ↑collagen content in BPO patients with respect to controls • ↑collagen content in patients with severe symptoms
Horn, 2004 [44]	Observational	54	• Correlation between abnormal morphology and impaired bladder compliance and decreased capacity
Collado, 2006 [11]	Case control	33	• ↑ intrafascicular and interfascicular collagen • ↑ intrafascicular fibrosis in BOO patients with history of AUR • ↑ SMCs diameter in BOO patients with respect to controls • Not significant differences in terms of SMCs diameter between BOO patients with and without AUR history • Positive correlation between SMCs diameter and symptoms duration
Rubinstein, 2007 [18]	Case control	10	• ↑ collagen and elastic fibers
Blatt, 2012 [16]	Case control	17	• Poor post-TURP voiding outcome in patients with detrusor ultrastructural pattern characterized by variable muscle cell size, muscle cell shape, abnormal fascicle arrangement and collagenosis.
Bellucci, 2017 [21]	Case control	19	• ↑ C/M • Significant negative correlation between C/M and bladder compliance • Significant correlation between the probability of urinary retention and C/M
Averbeck, 2017 [20]	Observational	38	• ↑ C/M in patients with PVR ≥ 200 mL and in those with reduced bladder compliance

*AUR* acute urinary retention, *BOO* bladder outlet obstruction, *C/M* connective tissue-to-smooth muscle ratio, *PVR* post void residual volume, *SMCs* smooth muscle cells, *TURP* trans urethral resection of the prostate

### SMCs alterations

Detrusor SMCs hypertrophy represents the most relevant morphological alteration occurring in BOO patients. Gilpin et al. compared the morphological and morphometric characteristics of detrusor specimens from patients with unequivocal urodynamic BOO accompanied by evidence of severe bladder trabeculation at cystoscopy and subjects with normal urodynamic assessment and absent trabeculation [10]. Although authors did not find morphological evidences of SMCs hyperplasia, the mean profile area of SMCs was higher in BOO patients due to hypertrophy. Similarly, Collado et al. demonstrated that detrusor SMCs diameter was significantly higher in BOO patients with respect to controls [11]. Authors found a positive correlation between SMCs diameter and symptoms duration but no statistically significant differences between BOO patients with and without history of Acute Urinary Retention (AUR). Electron microscopy studies also confirmed the evidence of SMCs hypertrophy in many cases. Elbadawi et al. investigated the ultrastructural basis of obstructive detrusor dysfunction in a prospective case-control study enrolling 35 elderly subjects [12]. Authors described the “myohypertrophy” structural pattern in patients with urodynamic proven BOO. This pattern was characterized by 4 distinctive features, including SMCs hypertrophy, marked widening of intercellular spaces with reduced normal intermediate cell junctions, increased deposition of collagen and elastin between SMCs, and patchy distribution of the preceding features in various muscle fascicles. This ultrastructural pattern was confirmed by other authors in patients with urodynamic evidence of BOO [13, 14]. Of note, the evidence of SMCs hypertrophy has not been confirmed in all patients. In their light microscopy study, Gosling et al. failed to found evidences of SMCs hypertrophy or hyperplasia in a subset of patients with unequivocal BPO accompanied by trabeculated urinary bladder [15]. On the contrary, authors identified some muscle bundles containing SMCs characterized by a small diameter [15]. Elbadawi et al. showed degeneration of SMCs in the specimens of patients who had impaired detrusor contractility [12]. The degenerative pattern was confirmed by Brierly et al. in BOO patients with high post void residual volume [14].

### ECM alterations

Detrusor ECM remodeling is characterized by increased accumulation of collagen and elastic fibers in both the interfascicular and intrafascicular compartments. This finding has been confirmed in several studies using both light and electron microscopy techniques [10, 11, 15–21]. Interestingly, Gilpin et al. found that patients with evidence of interfascicular connective tissue infiltration had the highest levels of SMCs mean profile area thus leading the authors to hypothesize that the deposition of connective tissue occurs at a later stage with respect to onset of SMCs hypertrophy [10]. This hypothesis has been confirmed by other studies. Inui et al. investigated the relationship between the amount of detrusor connective tissue in patients with BPH and the degree of bladder hypertrophy evaluated by ultrasound estimated bladder weight. In detail, authors compared the ratio of connective tissue-to-smooth muscle (C/M) between controls and BPE cases [19]. The study failed to found statistically significant differences between the two groups (27.3% in BPE patients and 24.7% in controls). However, a significant positive linear relationship between C/M and estimated bladder weight was evident in all BPE patients with estimated bladder weight ≥ 60 g [19]. Interestingly, 30% of BPE patients had C/M < 20% compared to only 7.7% of controls. Author hypothesized that the increase in ultrasound estimated bladder weight is caused at early stages by hyperplasia and/or hypertrophy of detrusor SMCs leading to a lower C/M and later to the additional increase of connective tissue with higher C/M. Other studies confirmed the occurrence of increased detrusor ECM accumulation with more advanced stages within the natural history of BOO. Collado et al. compared detrusor C/M of patients with urodynamic BOO and no history of AUR, patients with urodynamic BOO and history of AUR and non-obstructed controls [11]. Patients with BOO (obstruction and AUR groups) had a significantly higher intrafascicular and interfascicular collagen content than the control group. Patients with history of AUR had statistically significant higher levels of intrafascicular collagen than BOO patients without history of AUR. Moreover, authors found a statistically significant correlation between the amount of intrafascicular fibrosis and detrusor pressure at maximum urinary flow as well as with the Abrams-Griffiths number in BOO patients without history of AUR at pre-operative urodynamics. Additionally, a statistically significant negative correlation was found between intrafascicular fibrosis and postoperative bladder compliance in the same group of patients. Finally, a positive and significant correlation was found between intrafascicular fibrosis and both detrusor pressure at maximum urinary flow and the Abrams-Griffiths number in BOO patients with history of AUR at post-operative urodynamic evaluation [11]. Averbeck et al. evaluated the collagen content in the bladder wall of men undergoing open prostate surgery. Although BOO was not a predictor of increased collagen deposition, patients with reduced bladder compliance and those with a PVR ≥ 200 mL showed a significantly higher C/M [20]. Similarly, Bellucci et al. found a significant negative correlation between C/M and bladder compliance [21]. Moreover, the probability of urinary retention increased significantly with the C/M. Mirone et al. found higher detrusor collagen content in patients with BPO and severe symptoms with respect to patients with moderate symptoms [22]. Blatt et al. investigated the correlation between detrusor ultrastructural features of patients with urodynamic BOO or a hypocontractile detrusor and clinical outcomes after transurethral resection of the prostate [16]. Authors found that the morphological pattern characterized by variable SMCs size, SMCs shape, abnormal fascicle arrangement and collagenosis correlated with poor postoperative voiding outcome. Holm et al. investigated the correlation between ultrastructural findings and urodynamic parameters in patients with BOO [17]. SMCs hypertrophy, occurrence of abnormal cell junctions and configurations, variation in intercellular distances, and intracellular changes were investigated. The increase in intra- and interfascicular elastin was only parameter which was found to relate to the degree of obstruction in BOO patients.

### Alterations occurring in the detrusor muscle: molecular aspects and signaling pathways

Data about molecular aspects and signaling pathways involved in BOO-induced detrusor remodeling mainly derive from in vitro cell culture models of human bladder SMCs (HBSMCs) exposed to stressful stimuli such as cyclic mechanical stretch, increased hydrostatic (HP) or cyclic hydrodynamic pressure (CHP), and hypoxia. Further evidences derive from genetic and molecular studies on tissue specimens from BOO patients. Table 2 summarizes evidences from these studies.

**Table 2:** Summary of studies evaluating BOO-induced molecular alterations and related cellular events in human detrusor

Author, year	Experimental conditions	Molecular alteration	Cellular events
Backhaus, 2002 [34]	HBSMCs exposed to HP (0.3, 20 and 40 cm H_2_O) for 1, 3, 7 and 24 h	↓ MMP-1, 2, 9 after exposure to 20 cm H_2_O for 7 h ↑TIMP-1 after exposure to 40 cm H_2_O 3, 7 and 24 h	
Wang, 2013 [24]	HBSMCs exposed to HP (0, 20, 40, 60, 80 and 100 cm H_2_O) for 6, 12, 24 and 72 h	↓expression of the gap junction connexin 43 under HP > 60 cm H_2_O for 24 h or HP > 40 cm H_2_O for 72 h.	
Chen, 2012 [28]	HBSMCs exposed to CHP (0, 100, 200, and 300 cm H_2_O)	↑ SGK1 expression and activity	↑ proliferation in the 200 and 300 cm H_2_O groups
Chen, 2014 [32]	HBSMCs exposed to CHP (0, 100, 200, and 300 cm H_2_O)	↑ Skp2 expression and ↓ p27 expression under 200 and 300 cmH_2_O CHP	
Wu, 2012 [31]	HBSMCs exposed to CHP (static, 100, 200, and 300 cm H_2_O)	Ras-related C3 botulinum toxin substrate 1, mitogen-activated protein kinase kinase 1/2 and extracellular regulated protein kinases 1/2 activated by 200 and 300 cmH_2_O CHP	↑proliferation under 200 and 300 cmH_2_O CHP
Preis, 2015 [27]	HBSMCs exposed to HP of 136 cm H_2_O for 1 h	↑ expression of PDGFR α and β	↑ proliferation
Sun, 2016 [29]	HBSMCs exposed to CHP up to 200 cm H_2_O	↑ miR-3180-5p	↑ proliferation
Sun, 2017 [30]	HBSMCs exposed to CHP up to 200 cm H_2_O	↑ miR 4323	↑ proliferation
Lee, 2006 [25]	HBSMCs exposed to HP (40 cm H_2_O) and/or acetylcholine for 24 h	Activation of muscarinic receptors	↑ proliferation ↑ hypertrophy
Lee, 2008 [26]	HBSMCs exposed to acetylcholine in the presence or absence of HP (10, 20, and 40 cm H_2_O)	↑ M2 and M3 receptors expression	↑ proliferation ↑ hypertrophy
Yang, 2008 [23]	HBSMCs exposed to cyclic stretch with maximum of 15% strain magnitude at a frequency of 0.3 Hz for either 1 h or 24 h.	30 genes upregulated and 59 downregulated after 1 h exposure 59 genes upregulated and 27 downregulated after 24 h exposure	
Backhaus, 2002 [34]	HBSMCs exposed to HP (0.3, 20 and 40 cm H_2_O)	↓ MMP-1, 2, 9 ↑TIMP-1	
Liang, 2017 [33]	HBSMCs exposed to HP (100, 200, or 300 cm H_2_O) and/or acetylcholine	↑ IL-6, monocyte chemoattractant protein, and RANTES	
Galvin, 2004 [7]	HBSMCs exposed to 1% O_2_tension for 24, 48, 72, and 96 h	↑ HIF-1α ↑ VEGF ↑ p27^kip1^	↓ proliferation
Wiafe, 2017 [35]	HBSMCs exposed to 3% O_2_tension for 2, 24, 48, and 72 h	↑ HIF1α, HIF2α, and HIF3α ↑VEGF ↑TGFβ1 ↑CTGF ↑ collagens 1, 2, 3, 4 ↑ fibronectin ↑aggrecan ↑TIMP ↑ α-smooth muscle actin ↑vimentin, ↑desmin ↑TNFα, IL 1β, and IL 6 ↓ IL-10	
Boopathi , 2011 [38]	Bladder samples from subjects with BOO and controls	↑ expression of GATA-6 in cases ↓ Caveolin-1 expression	
Koritsiadis, 2008 [36]	Bladder samples from subjects scheduled for BPE-surgery and controls	↑ HIF-1α expression in stromal cells between muscle bundles and in connective tissue beneath the mucosal layer	
Barbosa, 2017 [37]	Bladder samples from subjects with obstructive BPE and controls	↑ collagens I and III ↓ MMP-9 and TIMP-1 ↑VEGF ↓ CD105	
Gheinani, 2017 [43]	Bladder samples from subjects with different states of urodynamic defined BOO-induced bladder dysfunction	Progressive increase in the number of altered mRNA and miRNAs from the detrusor overactive to the obstruction group to the underactive detrusor groups	

*BOO* bladder outlet obstruction, *BPE* benign prostatic enlargement, *CHP* cyclic hydrodynamic pressure, *CTGF* connective tissue transforming growth factor, *HBSMCs* human bladder smooth muscle cells, *HIF* hypoxia inducible factor, *HP* hydrostatic pressures, *IL* interleukin, *MMP* matrix metalloproteinases, *PDGFR* platelet derived growth factor receptor, *SGK1* serum-glucocorticoid regulated kinase 1, *Skp2* S-phase kinase-associated protein 2, *TGF* transforming growth factor, *TIMP* tissue inhibitor *of* metalloproteinases, *TNF* tumor necrosis factor, *VEGF* vascular endothelial growth factor

### Effects of cyclic stretch

Yang et al. investigated the effects of cyclic stretch on HBSMCs gene expression [23]. Authors identified multiple mechano-responsive genes encoding *cytokine, growth-related factors, adhesive molecules, signal transduction molecules, cytoskeleton and extracellular matrix proteins, developmental, differentiation, and inflammatory factors*. Twelve of proteins encoded by these genes had interacting partners in the vascular system and were functionally involved in multiple aspects of angiogenesis and vascular development such as endothelial cell proliferation and migration, SMCs differentiation, and arterial-venous differentiation.

### Effects of increased pressure

The effects of increased pressure on HBSMCs has been investigated by many authors. These studies demonstrated the existence of multiple pressure-dependent pathways involved into cellular processes such as adhesion, proliferation, inflammation, and ECM remodeling.

### Effects of increased pressure on cell adhesion

Wang et al. demonstrated a significant decreased expression of the gap junction connexin 43 under hydrostatic pressures > 60 cm H_2_O for 24 h or pressures > 40 cm H_2_O for 72 h [24].

### Effects of increased pressure on cell hypertrophy and hyperplasia

Lee et al. investigated the effects of HP on HBSMCs in terms of cell hypertrophy and hyperplasia and the potential role of muscarinic receptors [26]. HBSMCs proliferation and hypertrophy were measured by 3H-thymidine and leucine incorporation assays, respectively [25]. 3H-thymidine incorporation increased by 16.7, 25.9 and 39.4% after exposure to acetylcholine, 40 cmH_2_O HP, and both, respectively. Similarly, leucine incorporation increased by 66.5, 66.5 and 81.8%, after exposure to acetylcholine, 40 cmH_2_O HP, and both, respectively. These findings were consistent with increased proliferation and hypertrophy, respectively. Antimuscarinic agents determined a dramatic decrease in thymidine and leucine incorporation for cells exposed to increased HP, most pronounced when both M2 and M3 receptor antagonist were applied. In a subsequent study, authors found that M2 and M3 receptor expression increases in a time- and pressure-dependent manner in isolated HBSMCs [26]. Preis et al. investigated the role of platelet derived growth factor (PDGF) pathway in pressure-induced proliferation [27]. Exposure of HBSMCs to HP induced proliferation in a time dependent manner. Moreover, HBSMCs showed increased PDGF receptor (PDGFR) α and β expression. Interestingly, DNA synthesis in cells with intact PDGFR α was increased after short-term HP but cells lacking PDGFR α did not proliferate. All studies evaluating the effects of CHP on HBSMCs demonstrated increased proliferation under CHP > 100 cmH_2_O. Involved signaling pathways include: phosphoinositide 3-kinase/serum-glucocorticoid regulated kinase 1, S-phase kinase-associated protein 2, p27, Ras-related C3 botulinum toxin substrate 1, miR-3180-5p and miR 4323 [28–32]. MiR-3180-5p promotes HBSMCs proliferation by the activation of the pro-proliferative cyclin-dependent kinase 2 pathway [29]. On the other hand, miR 4323 can promote HBSMCs proliferation by inhibiting LYN expression and activating the Erk1/2 pathway, also known as the mitogen activated protein kinase signaling pathway [30].

### Effects of increased pressure on inflammatory pathways

Liang et al. investigated the effects of HP and acetylcholine on the release of inflammatory cytokines in HBSMCs to test the hypothesis that mechanical force and muscarinic receptors have pro-inflammatory effect in obstructed bladder [33]. HP produced a significant time-dependent and pressure-dependent increase in expressions of inflammatory genes. HP of 200 cm H_2_O for 24 h was associated with a statistically significant increase of monocyte chemoattractant protein, IL-6, and RANTES. Both NFκB and ERK1/2 pathways were proved to be involved in pressure-induced inflammation.

### Effects of increased pressure on ECM remodeling

Backhaus et al. applied HP to HBSMCs to determine the effect on matrix metalloproteinases (MMPs) and tissue inhibitors of metalloproteinases (TIMP) [34]. Exposure of HBSMCs to a sustained HP of 20 cm H_2_O for 7 h resulted in a significant time dependent decrease in MMP-1, 2 and 9 activities compared to controls maintained at atmospheric pressure. TIMP-1 levels increased an average of 10% after exposure to 20 cm H_2_O. These changes became statistically significant when the cells were exposed to 40 cm H_2_O.

### Effects of hypoxia

Available evidences demonstrate that hypoxia can modulate signaling pathways involved in angiogenesis, proliferation and ECM remodeling.

### Effects of hypoxia on hypoxia inducible factor and vascular endothelial growth factor

Galvin et al. demonstrated significant time-dependent upregulation of hypoxia inducible factor (HIF)-1α and vascular endothelial growth factor (VEGF) in HBSMCs exposed to hypoxia [7]. HIF-1α expression was maximal at 72 h while a twofold increase in VEGF production was evident after 24 h of hypoxia and this increase continued in a time-dependent manner. Wiafe et al. investigated the effects of hypoxia on HIF1α, HIF2α, HIF3α and VEGF expression [35]. Transcription of HIF1α and HIF2α demonstrated a time-dependent increased expression and were transiently upregulated in response to short-term hypoxia (2–24 h). HIF3α genes and protein were significantly expressed after 72 h of hypoxia when HIF1 and HIF2α proteins had resumed normoxic control levels. VEGF mRNA increased significantly after 24 and 72 h. The up-regulation of HIF and VEGF has been confirmed in bladder specimens from LUTS/BPE subjects. Koritsiadis et al. compared the expression of HIF-1alpha and carbonic anhydrase IX, a further cellular marker of hypoxia, in detrusor tissue retrieved from patients scheduled for surgery to treat BPE and controls [36]. The mean number of total cells immunoreactive to HIF-1α in the study group was significantly higher with respect to the control group. HIF-1α was expressed mainly in stromal cells between muscle bundles and in connective tissue beneath the mucosal layer, while urothelium and detrusor had no immunoreactivity [36]. Interestingly, the HIF-1α response was limited in a time-dependent manner. Indeed, the probability of HIF-1α immunoreactivity was four times greater in men with BOO for < 10 years, than in those with BOO for > 10 years with an odds ratio of 4.25 thus suggesting that the bladder can compensate for the first few years after that the adaptive response declines [36]. Moreover, the risk of identifying a high expression of HIF-1α was four times higher in patients with urinary retention [36]. Barbosa et al. compared the gene expression of the angiogenic growth factor VEGF in bladder specimens from patients with obstructive BPH with grade IV or higher BOO as per Schäfer criteria and age-matched controls [37]. Patients with BOO presented a statistically significant overexpression of VEGF. Interestingly, upregulation of VEGF was particularly evident in subjects with risk factors for atherosclerosis.

### Effects of hypoxia on HBSMCs proliferation

Galvin et al. demonstrated that HBSMCs exposed to hypoxia maintain their cell viability in culture and do not undergo cell death when placed under hypoxic conditions [7]. However, hypoxia significantly reduces the rate of proliferation in a time-dependent manner, associated with an increase in the cell cycle inhibitor p27^kip1^ [7].

### Effects of hypoxia on HBSMCs differentiation

Wiafe et al. found evidences in favor of HBSMCs dedifferentiation, as demonstrated by the increased expression of α-smooth muscle actin, vimentin, and desmin, and acquisition of a profibrotic phenotype [35]. Pro-fibrotic changes included the upregulation of SMAD 2, SMAD 3, and connective tissue growth factor (CTGF) genes as well as collagens 1, 2, 3, and 4, fibronectin, aggrecan, and TIMP-1 transcripts [35]. Collagen 1 transcripts exhibited a consistent increase over the entire time course, with a 3.4-fold increase after 2 h eventually reaching a maximum fold increase of 12 by 72 h. Collagen 2 transcript levels showed a 4-fold increase by 72 h; collagen 3 exhibited a similar increase, although a 5-fold increase was evident at 48 and 72 h. Collagen 4 transcripts rose by almost 8-fold following 72 h hypoxia. Total secreted collagen remained at control values until 24 h at which point levels rose by 100% and values remained consistently elevated during prolonged hypoxia. Fibronectin transcripts showed a consistent increase from 1.9 to 3.9-fold for values between 24 and 72 h.

### Effects of hypoxia on inflammatory pathways

Wiafe et al. demonstrated that hypoxia can produce a robust inflammatory response in isolated HBSMCs [35]. Indeed, hypoxia induced increased expression of TNFα, IL 1β, and IL 6 which are all part of the acute phase proteins secreted in response to inflammation. Transcript levels of the anti-inflammatory cytokine, IL-10 exhibited a consistent decline [35].

### Other SMCs molecular alterations

Boopathi et al. demonstrated the loss of caveolin-1, a protein that has a pivotal role in regulating SMCs contractile activity, in bladder wall smooth muscle from BPO subjects [38].

### Alterations occurring in the neuronal compartment

We identified four studies describing BOO-induced morphological alterations involving bladder innervation and two studies investigating the potential role of tissue nerve growth factor (Table 3). Golsin et al. found a 56% reduction in the number of acetylcholine-positive nerves in the specimens from obstructed bladder with respect to controls [39]. Cumming et al. confirmed the significant reduction of detrusor innervation in bladder biopsies from patients with urodynamic confirmed BOO [40]. Interestingly, innervation level was normalized in 80% of patients after BOO relief. Chapple et al. demonstrated a reduction in the density of innervation by vasoactive intestinal polypeptide, calcitonin gene-related peptide, substance P and somatostatin-immunoreactive but not neuropeptide Y-immunoreactive nerve fibers in bladder specimens from BOO patients [41]. Elbadawi et al. showed axon degeneration at electron microscopy in specimens of patients who had impaired detrusor contractility [12]. The role of tissue nerve growth factor, a key signal in the regulation of nerve physiology, is controversial. Steers et al. found that the amount of nerve growth factor in grossly hypertrophied human bladders exceeded that in non-hypertrophied samples [42]. In the study by Barbosa et al. the levels of nerve growth factor and of nerve growth factor receptor in bladder tissue from the overall BPO population were not different with respect to controls [37]. However, the levels of nerve growth factor receptors were higher in the subgroup of smokers and dyslipidemic BPO patients [37].

**Table 3:** Summary of studies evaluating BOO-induced neuronal alterations

Author, year	Study design	Subjects in the cases group (n)	Findings
Gosling, 1986 [39]	Case-control	19	↓ autonomic nerve supply
Chapple, 1992 [41]	Case-control	19	↓ density of innervation by vasoactive intestinal polypeptide, calcitonin gene-related peptide, substance P and somatostatin immunoreactive nerve fibers in the obstructed bladder.
Cumming, 1992 [40]	Case-control	10	↓innervation
Elbadawi, 1993 [12]	Case control	7	↑axon degeneration in patients with impaired detrusor contractility
Steers, 1991 [42]	Case-control	–	↑ NGF in grossly hypertrophied human bladders
Barbosa, 2017 [37]	Case-control	43	↑ NGF receptor expression in smokers and dyslipidemic patients

*NGF* nerve growth factor

## Discussion

**Fig. 2:**
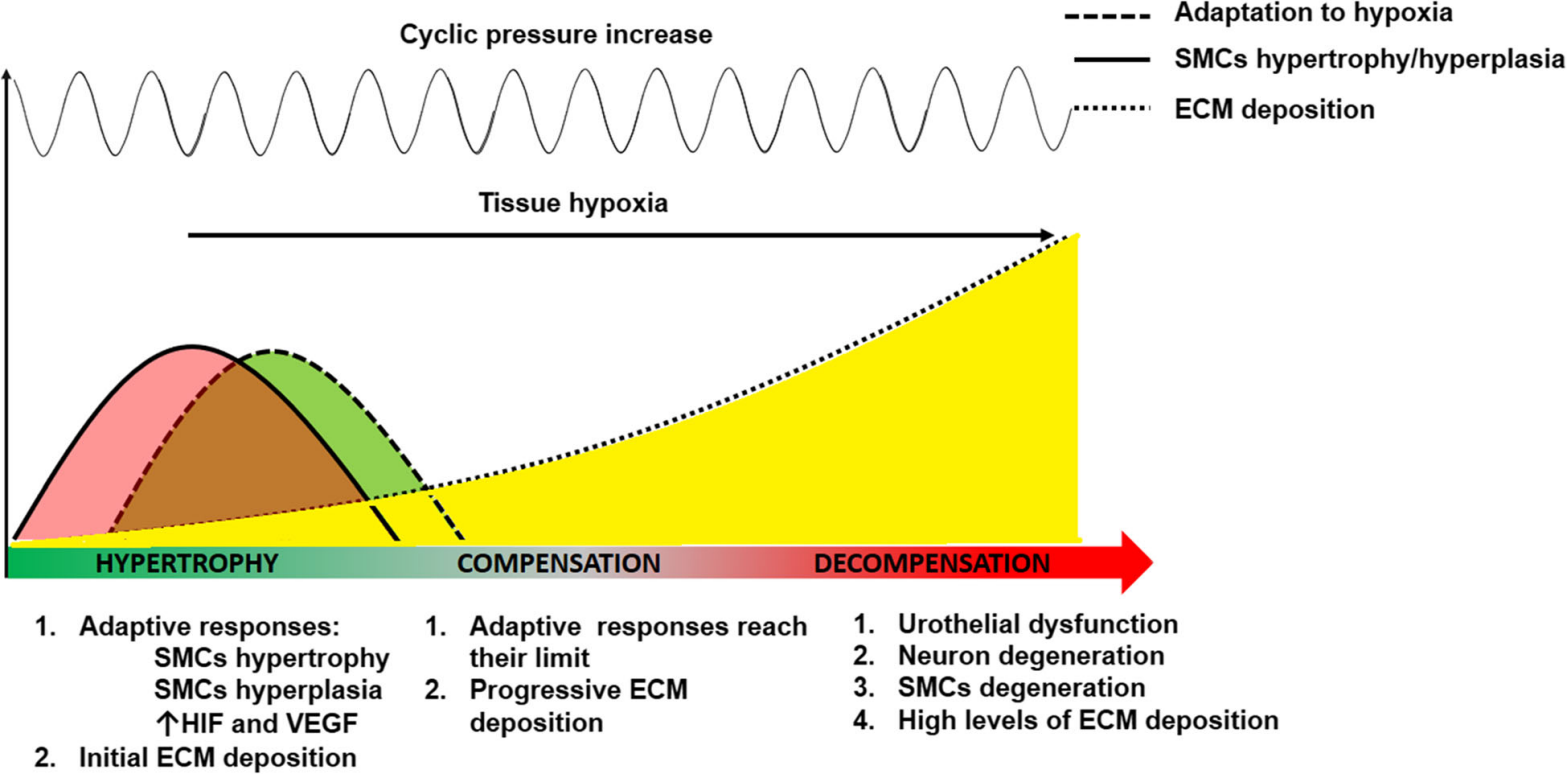
Proposed three-stage model for BOO-induced bladder remodeling in humans

To our knowledge, we performed the first systematic review of studies investigating BOO-induced morphological and molecular alterations in human bladder. Data summarized demonstrate the occurrence of a remodeling process involving multiple cellular compartments, namely urothelium, suburothelium, detrusor SMCs, detrusor ECM, and neurons. Based on evidences from in-vitro HBSMCs cultures, cyclical stretch, increased pressure and hypoxia can modulate several signaling pathways potentially involved in this process. Taken together, these data support the hypothesis that the natural history of BOO may be characterized, also in humans, by three morpho-functional stages: an initial hypertrophy phase, a subsequent compensation, and a late decompensation (Fig. 2). Increased intravescical pressure during bladder voiding, the pathognomonic urodynamic feature of BOO, can be considered the “primum movens”. Indeed, it can stimulate compensatory SMCs hypertrophy and proliferation as demonstrated by in vitro studies. Tissue hypoxia subsequently intervenes as further critical stress factor. It is due to the imbalance between increased oxygen demand and lower oxygen delivery and may arise early in the natural history of BOO-induced bladder remodeling. Clinically, detrusor hypoxia has been confirmed in studies on human subjects with evidence of BOO [45–47]. Compensatory responses to hypoxia have been demonstrated in several human tissues, including the bladder. These include hypoxia-induced pathways such as HIF and VEGF. Available data suggest that, similar to animal models, these adaptive responses may counteract hypoxia only for a limited period of time. Persistent hypoxia also inhibits HBSMCs proliferation thus favoring the transition from the hypertrophy to the compensation phase, and activates signaling involved in ECM remodeling and collagen accumulation that characterize the transition from compensation to decompensation. Indeed, increased deposition of collagen and elastin in the interfascicular and intrafascicular detrusor compartments can alter the bio-mechanical properties of the bladder causing decreased compliance and impaired voiding function, which are feature considered by some authors as clinical marker of decompensation. Results from the present review have relevant clinical implications. In recent years evidences have emerged demonstrating that LUTS/BPO represent a progressive disorder in many patients [48–50]. Progression, however, has been defined based on clinical parameters such as deterioration of symptoms and health-related quality of life, decreased urinary peak flow rate, increased prostate size, and unfavorable outcomes such as AUR and BPE-related surgery [48–50]. Based on the results from the present study, clinicians should be aware of the fact that, beyond subjective symptoms, BPO also causes progressive morphological remodeling of the bladder with potential serious functional impairments. Consequently, this aspect should be carefully taken in account in the management of these patients and therapeutic outcomes should include not only the improvement of subjective symptoms but also the prevention of pathologic bladder remodeling. Evidences summarized in the present review suggest the existence of multiple signaling pathways that can represent potential targets for future therapies. Meantime, increased bladder pressure is the only pathophysiological mechanism that can be realistically improved in everyday clinical practice. Although multiple treatment options are available to treat LUTS/BPO, only surgery, alpha-1 adrenergic antagonists and 5-alpha reductase inhibitors have been reported to improve BOO by reducing bladder pressures. The progressive model of bladder remodeling emerged from evidences summarized in the present study also contributes to explain the failures of surgical and medical therapies when prescribed later in the natural history of BOO and suggests to intervene early [48–50]. Although adherence to medical therapy for LUTS/BPO has been reported to prevent clinical disease progression the advantages in terms of morphological remodeling deserve further investigations [51]. At time, only few clinical markers of morphological bladder remodeling are available including increased estimated bladder weight and endoscopic evidence of bladder trabeculation. The role of bladder biopsies is controversial [44]. Results from the present study provide the basis for future investigations about urinary and/or serum markers of bladder remodeling. Some limits of the present study should be acknowledged: studies included are often outdated and enrolled a limited number of patients. Cultured cells may differ from fresh detrusor tissues as potential interactions among various compartments are not considered and cells are simply exposed to a single stress factor. Consequently, the molecular mechanism of bladder remodeling in BOO remains unclear and it remains difficult to establish an integrated signaling pathway. Further issues deserving investigations are the timing and the reversibility of BOO-induced bladder remodeling.

## Conclusions

Evidences from available studies on human tissues demonstrate that BOO induces molecular and morphological alterations in multiple bladder compartments, namely urothelium, suburothelium, detrusor SMCs, detrusor ECM, and neurons. Cyclic stretch, increased pressure and hypoxia have been demonstrated to modulate multiple signaling pathways involved in these processes. A three-stages model can be hypothesized to characterize BOO-induced bladder remodeling also in humans: hypertrophy, compensation, decompensation.

## Abbreviations

AUR: Acute urinary retention
BOO: Bladder outlet obstruction
BPE: Benign prostatic enlargement
BPO: Benign prostatic obstruction
C/M: Connective Tissue-to-Smooth Muscle Ratio
CHP: Chronic hydrostatic pressure
CTGF: Connective tissue growth factor
ECM: Extracellular matrix
HBSMCs: Human bladder smooth muscle cells
HIF: Hypoxia inducible factor
HP: Hydrostatic pressure
IL: Interleukin
LUTS: Lower urinary tract symptoms
MMPs: Matrix metalloproteinases
PDGF: Platelet derived growth factor
SMCs: Smooth Muscle Cells
TIMP: Tissue inhibitors of metalloproteases
TNF: Tumor necrosis factor
VEGF: Vascular endothelial growth factor
